# Chromatin structure and gene transcription of recombinant p53 adenovirus vector within host

**DOI:** 10.3389/fmolb.2025.1562357

**Published:** 2025-02-28

**Authors:** Duo Ning, Yuqing Deng, Simon Zhongyuan Tian

**Affiliations:** Department of Systems Biology, School of Life Sciences, Southern University of Science and Technology, Shenzhen, Guangdong, China

**Keywords:** Ad-p53, ChIA-PET, p53, colon cancer, chromatin interaction, 3D genome

## Abstract

**Introduction:**

The recombinant human p53 adenovirus (Ad-p53) offers a promising approach for cancer therapy, yet its chromatin structure and effects on host chromatin organization and gene expression are not fully understood.

**Methods:**

In this study, we employed *in situ* ChIA-PET to investigate the colorectal cancer cell line HCT116 with p53 knockout, comparing them to cells infected with the adenovirus-vector expressing p53. We examined alterations in chromatin interactions and gene expression following treatment with the anti-cancer drug 5-fluorouracil (5-FU).

**Results:**

Our results indicate that Ad-p53 forms a specific chromatin architecture within the vector and mainly interacts with repressive or inactive regions of host chromatin, without significantly affecting the expression of associated genes. Additionally, Ad-p53 does not affect topologically associating domains (TADs) or A/B compartments in the host genome.

**Discussion:**

These findings suggest that while Ad-p53 boosts p53 expression, enhancing drug sensitivity without substantially altering host HCT116 chromatin architecture.

## 1 Introduction

Cancer is a major cause of global mortality, and gene therapy has emerged as a promising treatment option for various diseases, including cancer (Dunbar et al., 2018; Mohd Abas et al., 2024). Adenovirus-based gene transfer vectors are frequently used in clinical gene therapy trials due to their high efficiency in delivering genes to a wide range of cell types (Sato-Dahlman et al., 2020; Park and Lee, 2024). Adenovirus vectors typically have an extrachromosomal life cycle, enabling transient expression of the delivered gene without integrating into the host genome (Stephen et al., 2010). The recombinant human p53 adenovirus (Ad-p53), known as Gendicine, became the first gene therapy product for cancer approved for clinical use in China in 2003 (Pearson et al., 2004; Wilson, 2005). The Ad-p53 vector contains the wild-type *TP53* gene, which encodes the p53 protein. p53 is a critical tumor suppressor that regulates numerous genes involved in apoptosis, DNA repair, and cell cycle arrest, thereby effectively inhibiting tumor progression (Hafner et al., 2019). However, the *TP53* gene is one of the most frequently mutated genes in cancer (Liu et al., 2024), and mutated p53 contributes to tumor development and proliferation (Chen et al., 2022; Kennedy and Lowe, 2022). Restoring the function of wild-type p53 is therefore a crucial therapeutic goal (Peuget et al., 2024). Ad-p53 works by delivering wild-type p53 to tumor cells using an adenoviral vector, thereby restoring its tumor-suppressive function. Several studies have summarized the effectiveness of this approach in cancer therapy (Zhang et al., 2018; Hosmani et al., 2021).

Adenoviral vectors have emerged as promising platforms for cancer therapy; however, the safety of gene therapy remains a significant concern (Tang and Xu, 2020). While extensive research has investigate viral genome replication and cellular processes (Charman et al., 2019), critical questions remain regarding the detailed mechanisms of virus-host interactions, particularly how these interactions affect chromatin architecture of vector and host, an area that remains largely unexplored. Recent studies have demonstrated that, in addition to encoding proteins that regulate host gene expression, viruses can interact with host chromatin, remodeling its structure and affecting gene expression (Moreau et al., 2018; Kim and Lieberman, 2024; Venu et al., 2024).

Host chromatin can be organized into a hierarchical three-dimensional (3D) architecture, which is crucial for regulating gene expression. Chromatin is organized into chromosomal territories, further partitioned into A/B compartments, topologically associating domains (TADs), and chromatin loops (Kempfer and Pombo, 2020). Advances in 3D genomic techniques, such as High-through chromosome conformation capture (Hi-C) (Lieberman-Aiden et al., 2009) and Chromatin interaction analysis by paired-end tag sequencing (ChIA-PET) (Fullwood et al., 2009), have greatly enhanced our understanding of chromatin organization. Hi-C captures all chromatin interactions throughout the nucleus and is suitable for studying higher-order structures, while ChIA-PET provides high-resolution insights into chromatin interactions mediated by specific proteins, elucidating their roles in genome organization and gene regulation. The exploration of chromatin structure allows us to better understand how specific structural features facilitate precise interactions between regulatory elements to control gene expression (Han et al., 2024). Disruptions in these structures, such as TADs, may lead to aberrant enhancer-promoter interactions and the activation of oncogenes (Valton and Dekker, 2016). Viral infections can also induce refolding of both viral and host genomes, disrupting gene expression and potentially contributing to oncogenic transformation (Kim and Lieberman, 2024). Therefore, exploring the interaction between Ad-p53 and the host genome within the context of 3D chromatin structure is essential for understanding the molecular mechanisms underlying these processes.

To explore the interaction between Ad-p53 and the host, as well as its effect on host chromatin structure and gene regulation, we used the colorectal cancer cell line HCT116, which expresses wild-type (WT) p53, and its p53 knockout (KO) variant. p53 was stabilized and activated by treating the cells with the anti-cancer drug 5-fluorouracil (5-FU) (Longley et al., 2003), as p53 levels are normally regulated by MDM2-mediated ubiquitination and proteasomal degradation (Haupt et al., 1997; Kubbutat et al., 1997). We employed p53 ChIA-PET to examine p53-associated chromatin interactions in both the vector and host genomes, and used RNA polymerase II (RNAPII) ChIA-PET to investigate RNAPII-associated enhancer-promoter chromatin interactions involved in gene regulation. Finally, we assessed the impact of Ad-p53 infection on gene expression through transcriptome RNA sequencing (RNA-seq).

Our results demonstrate that p53 and RNAPII mediate extensive chromatin interactions within the Ad-p53 vector, resulting in the formation of a distinct 3D structure characterized by three prominent structural blocks. Moreover, p53-mediated interactions between Ad-p53 and host chromatin predominantly occur in repressive or transcriptionally inactive regions of the host genome. Consistent with this, RNA-seq analyses revealed that these interactions did not significantly affect the expression of Ad-p53-associated host genes. Additionally, analyses of A/B compartments and TAD structures indicated that Ad-p53-host interactions do not disrupt the higher-order chromatin architecture of the host genome.

These findings provide compelling evidence that, although Ad-p53 interacts with host chromatin, it does not alter host gene expression or chromatin organization. Our study underscores the safety and efficacy of Ad-p53 as a therapeutic tool and advances our understanding of how adenoviral vectors function within the nuclear environment to regulate gene expression and chromatin structure.

## 2 Materials and methods

### 2.1 Cell culture

Human colon cancer cell line HCT116 (HCT116 p53^+/+^) and its derived isogenic p53 knock out cell line (HCT116 p53^−/−^) were obtained from Professor Zhang Weimin’s lab. Cells were cultured in DMEM (Thermo Fisher, 11965092) supplemented with 10% fetal bovine serum (FBS, Excell Bio, FSP500), 100 U/mL penicillin, and 100 μg/mL streptomycin (Thermo Fisher, 15140122) at 37°C in a humidified atmosphere.

### 2.2 Ad-p53 and 5-FU treatment

Ad-p53 was purchased from Shenzhen SiBiono GeneTech under the commercial name Gendicine (Zhang et al., 2018), with the lot number SJ202101. The batch inspection report confirms that the viral concentration for this lot is 4.7 × 10^10^ VP/ml. Ad-p53 was diluted in DMEM to a final concentration of 1 × 10^8^ VP/mL and pre-warmed to 37°C before use. HCT116 p53 KO cells (2 × 10^6^) were seeded to 15 cm plate, and 24 h post-passage, they were incubated with 10 mL of 1 × 10^8^ VP/mL Ad-p53 (500 MOI) for 2 h at 37°C. This MOI was selected based on previous reports (Sun et al., 2014) regarding Ad-p53 testing in colon cancer cells, where the p53 ChIP efficiency at 500 MOI was comparable to that observed in wild-type (WT) cells. The medium was then supplemented with 20 mL of DMEM containing 15% FBS (final FBS concentration: 10%) and further incubated at 37°C for 61 h. The 61-h treatment period was chosen based on a previous report (Zhang et al., 2018), which indicates that Gendicine (Ad-p53) expression requires approximately 72 h to achieve peak efficacy. To ensure optimal infection and expression, we allowed the cells to attach for 24 h before adding Ad-p53. After a 2-h incubation with Ad-p53 in fresh DMEM (without FBS), the medium was supplemented with DMEM containing 15% FBS and further incubated at 37°C for an additional 61 h. Following this 61-h incubation, cells were either treated with 5-FU for 9 h or left untreated as controls, resulting in a total of 72 h post-Ad-p53 treatment, aligning with the reported peak efficacy. Additionally, we optimized cell density to maintain 80%–90% confluence at the time of harvest, preventing overconfluence, which could otherwise reduce the effectiveness of 5-FU treatment by affecting drug accessibility.

5-FU (Sigma, F6627-1G) was dissolved in DMSO (Sigma, D2650-100ML) as a stock solution at 375 mM. HCT116 WT and HCT116 p53 KO cells (with or without Ad-p53 infection) were treated with 375 μM 5-FU for 9 h or left untreated as controls.

### 2.3 RNA-seq libraries generation

After Ad-p53 and 5-FU treatment, cells were collected in 1.5 mL tubes containing TRIzol (Thermo Fisher, 15596026) and stored at −80°C. RNA isolation, RNA-seq library construction, and sequencing were performed by Novogene. Total RNA was extracted using Phenol-chloroform method, and rRNA was removed from the sample with the Ribo-zero™ Kit (Epicentre, MRZH116). Stranded RNA-seq libraries were prepared using the NEBNext® Ultra™ Directional RNA Library Prep Kit for Illumina® (NEB, E7420L) according to the manufacturer’s protocol. RNA-seq libraries were sequenced as 150 bp paired-ends read using an Illumina NovaSeq 6,000 sequencer.

### 2.4 Cell cross-linking

HCT116 WT, HCT116 p53 KO, and Ad-p53-infected cells, treated with 5-FU for 0 or 9 h, were cross-linked with 1% formaldehyde (Sigma, 47608) for 20 min at room temperature. Crosslinking was quenched with glycine (Sigma, 50046) for 10 min. Cells were washed twice with DPBS (Thermo Fisher, 14190250) and further cross-linked with 2 mM EGS (Thermo Fisher, 21565) for 45 min. After quenching with glycine, cell pellets were washed twice with DPBS and stored at −80°C.

### 2.5 *In situ* ChIA-PET libraries generation

*In situ* ChIA-PET libraries were prepared using antibodies against RNAPII (BioLegend, 664906) and p53 (Santa Cruz Biotechnology, sc-126), following the *in situ* ChIA-PET protocol (Wang et al., 2021). Briefly, about 10 million dual-cross-linked cells were used for each experiment. Cells were lysed in 0.1% SDS (sodium dodecyl sulfate) cell lysis buffer at 4°C for 1 h with rotation, spun down at 2,500 rpm for 5 min at 4°C, and the supernatant was discarded. Pellets were resuspended in 0.55% SDS solution and incubated sequentially at room temperature (RT) for 10 min, 62°C for 10 min, and 37°C for 10 min. To neutralize SDS, 295 μL of nuclease-free water and 25 μL of 20% Triton X-100 (Acros Organics, 327371000) were added, followed by rotation at 37°C for 15 min. Nuclei were digested overnight with AluI enzyme (NEB, R0137L) under constant agitation. A-tailing was performed using Klenow fragment (NEB, M0212L) and dATP (Thermo Fisher, 18252015), followed by proximity ligation using a biotin-containing bridge linker and T4 DNA ligase (NEB, M0202S) at 16°C overnight under rotation. The chromatin was fragmented to 2–3 kb by sonication, followed by immunoprecipitation with anti-RNAPII or anti-p53 antibodies conjugated to Protein G beads (Thermo Fisher, 10004D) overnight at 4°C.

The enriched chromatin sample was decrosslinked and released from Protein G beads by incubation with proteinase K (Thermo Fisher, AM2548) at 65°C overnight. Tn5 tagmentation (Vazyme, TD501-01) was used to fragment ChIP DNA and add sequencing adapters simultaneously. Proximity-ligated DNA fragments containing bridge linkers were enriched using M280 streptavidin beads (Thermo Fisher, 11205D), followed by PCR amplification and size selection (200–600 bp) using Ampure XP beads (Beckman, A63881). Libraries were sequenced as 150 bp paired-end reads on an Illumina NovaSeq 6,000 platform.

### 2.6 ChIA-PET data processing

ChIA-PET paired-end FASTQ files generated from paired-end Illumina sequencing were analyzed using the ChIA-PIPE pipeline (Lee et al., 2020). The data were aligned to a combined reference genome consisting of hg38 and Ad-p53. Hi-C files were generated using the ChIA-PIPE pipeline, which incorporated Juicer (v1.19.01) (Durand et al., 2016b), and 2D contact heatmaps were visualized in Juicebox (v2.17.00) (Durand et al., 2016a).

To analyze interactions between hg38 and Ad-p53, as well as intra-hg38 interactions, PETs with a genomic span of >8 kb and an extension of 500 bp were used to generate clusters for more precise interactions. For intra-Ad-p53 interactions, PETs with a genomic span of >600 bp and an extension of 0 bp were utilized. The chromatin interactions and binding intensity were displayed in BASIC Browser (Lee et al., 2020).

### 2.7 RNA-seq data processing

The FASTQ files were first trimmed to remove adapters and then separately aligned to the hg38 human reference genome and Ad-p53 using the STAR (v2.7.10b) (Dobin et al., 2013). Gene-level abundance was quantified using the RSEM (v1.2.28) program (Li and Dewey, 2011), and differential expression analysis between the two groups was performed using the edgeR package in R (v4.4.0) (Robinson et al., 2010).

### 2.8 TAD boundary calling

The Hi-C files generated by ChIA-PIPE were converted to. cool format using hic2cool (v0.8.3). Subsequently, hicFindTADs (v3.7.5) was utilized to identify TADs at resolutions of 25 kb, 50 kb, and 100 kb, applying a significance threshold of 0.05 (Ramírez et al., 2018).

### 2.9 A/B compartment identification

Juicer Tools (v1.19.01) was utilized to extract KR-normalized eigenvectors from Hi-C files at a resolution of 500 kb (Durand et al., 2016b).

## 3 Results

### 3.1 p53 and RNAPII mediated extensive chromatin interactions in Ad-p53 vector

**FIGURE 1:**
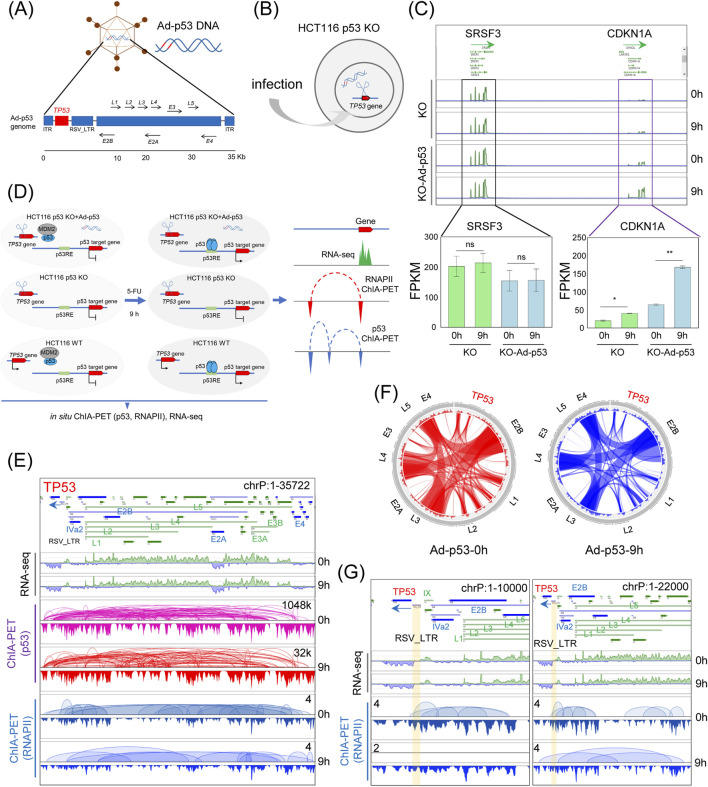
Mapping the 3D epigenome organization in Ad-p53-infected cells with inducible p53. **(A)** Schematic of the Ad-p53 vector; **(B)** Diagram of Ad-p53 transfection in HCT116 p53 KO cells; **(C)** Screenshots of BASIC browser views of *CDKN1A* and *SRSF3* gene expression in HCT116 p53 KO cells after Ad-p53 and 5-FU treatment (top), with corresponding FPKM values (bottom). T-test, ns: p > 0.05, not significant, *p < 0.05, **p < 0.01; error bars indicate the standard deviation (SD). 0 h: 5-FU untreated; 9 h: 5-FU treated for 9 h; **(D)** Schematic diagram of the experimental design; **(E)** Screenshots of BASIC browser views of gene expression, p53 and RNAPII binding, and associated chromatin interactions mediated by p53 and RNAPII within Ad-p53 vector; **(F)** Circos plots of p53-associated chromatin interactions within Ad-p53 vector, with or without 5-FU treatment for 9 h (9h and 0 h); **(G)** Screenshots of zoomed-in BASIC browser views of RNAPII-associated interactions at the *TP53* locus in the Ad-p53 vector.

To investigate the impact of the Ad-p53 vector on host genome organization and gene regulation, we infected HCT116 p53 KO cells (Figures 1A, B) with Ad-p53 and activated p53 expression by treating the cells with 5-FU for 9 h. RNA-seq analysis revealed that 5-FU treatment after Ad-p53 infection significantly upregulated the p53 target gene *CDKN1*A (Figure 1C), compared to both uninfected and untreated controls. The neighboring untargeted gene *SRSF3* showed no significant changes, confirming that our experimental conditions effectively restored p53 activity.

Based on the tested experimental conditions, we set up three experimental groups: p53 KO cells, p53 KO cells infected with Ad-p53, and WT cells. Each group was further subdivided into 5-FU-treated (for 9 h) and untreated controls. We then prepared p53-and RNAPII-ChIA-PET libraries, as well as RNA-seq libraries, for these treatments (Figure 1D).

RNA-seq results confirmed the transcription of *TP53* and other genes in the Ad-p53 vector, further validating successful Ad-p53 expression (Figure 1E). ChIA-PET analysis showed p53 binding across the Ad-p53 vector, mediating extensive chromatin interactions within the vector. These findings suggest that p53 is not only expressed from the Ad-p53 vector but also contributes to its structural reorganization. Notably, interactions between loci within the Ad-p53 vector, such as *E2B*, *E4*, *L5*, *L3*, and *L4*, were consistently maintained both before and after 5-FU treatment (Figure 1F). This indicates that p53 activation does not significantly alter the 3D structure of the Ad-p53 vector.

In addition to p53, RNAPII also binds to the Ad-p53 vector and mediates chromatin interactions within the vector. Notably, the RSV_LTR region serves as the promoter for the *TP53* gene within the Ad-p53 vector. Focusing on the *TP53* region in RNAPII ChIA-PET data (Figure 1G), we observed that RNAPII mediates chromatin interactions from the *TP53* promoter to other loci, both in 5-FU-treated and untreated cells. This suggests that RNAPII may play a role in regulating Ad-p53 transcription and contributes to the structural folding of the Ad-p53 vector.

In summary, our results show that the Ad-p53 vector restores p53 activity in HCT116 p53 KO cells, with 5-FU treatment further activating p53. ChIA-PET and RNA-seq analyses reveal that p53 mediates chromatin interactions within the Ad-p53 vector, maintaining a stable 3D structure. Additionally, RNAPII binds to the vector, potentially contributing to its transcriptional regulation and structural organization. These findings highlight the interplay between p53, RNAPII, and the Ad-p53 vector in shaping its architecture and gene expression in host cells.

### 3.2 The 2D contact map of Ad-p53 vector reveals three structural blocks

Although we have analyzed DNA interactions within the Ad-p53 vector, the higher-order structure of the vector remains unclear. Since ChIA-PET experiments use antibodies to enrich for specific protein-mediated chromatin interactions, it has been shown that incorporating non-clustered tags, which represent singleton inter-ligation data, can reflect higher-order topological proximity, similar to Hi-C data (Tang et al., 2015; Lee et al., 2020). This approach enabled us to explore the higher-order structures of both the Ad-p53 vector and the host genome, as visualized through Hi-C data.

**FIGURE 2:**
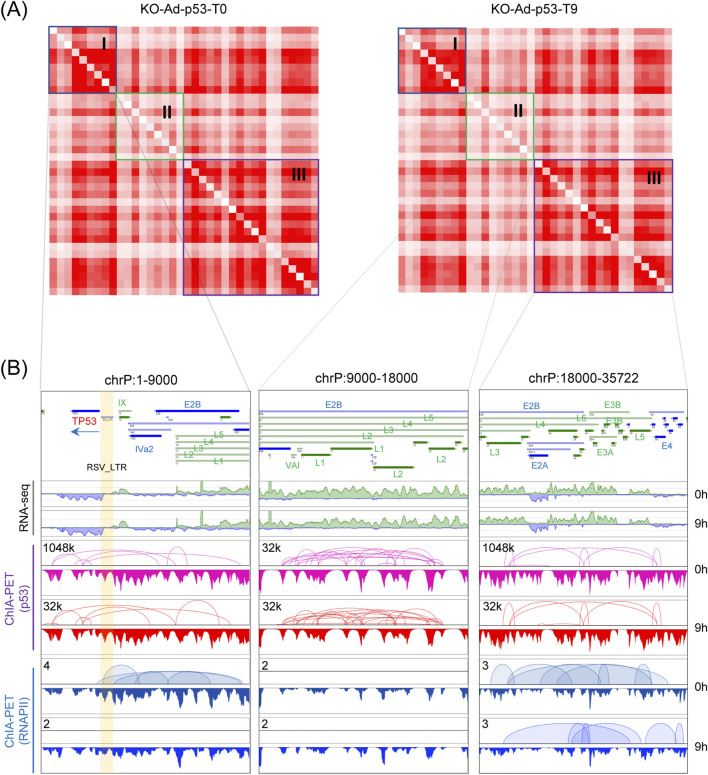
The 2D contact map of the Ad-p53 vector reveals three structural blocks. **(A)** 2D contact maps of p53 ChIA-PET data within Ad-p53 under 5-FU-treated (T9) and untreated (T0) conditions; **(B)** Screenshots of browser views of RNA-seq, p53 and RNAPII ChIA-PET results in three regions corresponding to the 2D contact maps shown in **(A)**.

We examined the 2D contact map of the Ad-p53 vector and identified three large blocks within the vector, which resemble typical TAD structures (Figure 2A). These structures were highly conserved even after 5-FU treatment, further validating the presence of a specific 3D organization within the Ad-p53 vector. The first block (1 bp to 9,000 bp) exhibited strong, localized interaction signals, indicating frequent chromatin interactions within this region. In contrast, the middle block (9,000 bp to 18,000 bp) showed weaker and more dispersed interaction signals, suggesting a more loosely organized structure. The final block (18,000 bp to 35,722 bp) displayed relatively high-frequency chromatin interactions, with clear boundaries separating it from adjacent regions.

We further investigated the p53-and RNAPII-mediated chromatin interactions within these three blocks (Figure 2B). The *TP53* gene, located in the first block (Block I), exhibited numerous p53-mediated chromatin interactions with regions such as *E2B*, *IVa2*, and the *L1-L5* genes. Notably, the early-expressed *E2B* gene, which is involved in viral replication, and *IVa2* that helps package viral DNA into immature virions (Christensen et al., 2008; Saha et al., 2014), both interacted with *TP53* gene. Additionally, the late-expressed *L1*-*L5* genes generally encode virion structural proteins (Scarsella et al., 2024), were also associated with *TP53* in the 3D structure. These findings suggest that p53 mediates interactions between the *TP53* gene body and these functionally significant genes, thereby influencing their organization in the 3D chromatin structure. In the Block II, most interactions were observed between the *L1*-*L5* and *E2B* gene bodies. In the third block (Block III), p53 further facilitated interactions between genes such as *E2A*, *L3*, *E4*, and *L4*, contributing to the overall 3D architecture of the Ad-p53 vector. Comparison between 5-FU untreated and treated samples revealed that these interactions were highly conserved.

In contrast to p53, RNAPII mediated fewer chromatin interactions within the Ad-p53 vector. Overall, the 2D contact map of the Ad-p53 vector revealed three TAD-like structures, with chromatin interactions primarily occurring within these blocks. Further analysis highlighted p53-mediated conserved interactions within each block.

### 3.3 Ad-p53 does not alter host gene expression via interaction with host chromatin

**FIGURE 3:**
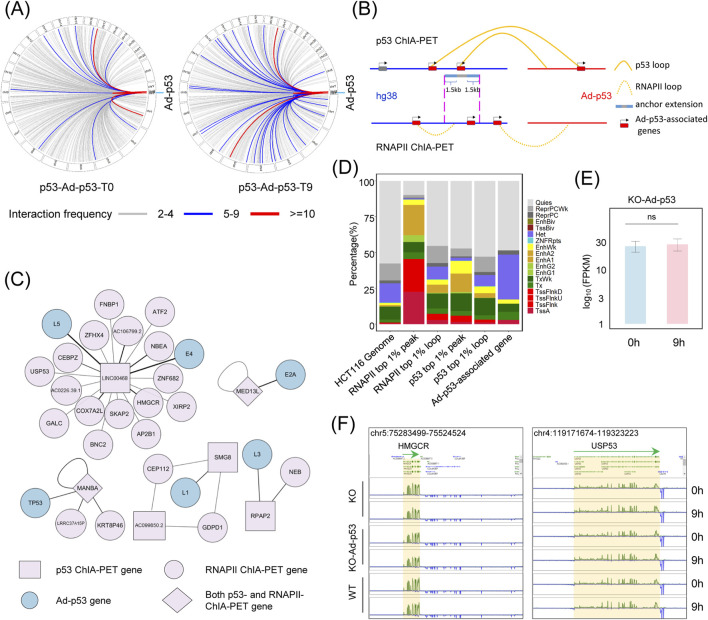
Ad-p53 does not alter host gene expression via interaction with host chromatin. **(A)** p53-associated interaction regions between Ad-p53 and host chromatin; **(B)** Diagram of the analysis of Ad-p53-associated genes through p53 and RNAPII-mediated chromatin interactions; **(C)** The network of genes involved in p53 and RNAPII-mediated interactions between Ad-p53 and host chromatin in HCT116 p53 KO cells after Ad-p53 infection and 5-FU treatment; **(D)** Chromatin states of host genome regions interacting with Ad-p53, which are related to Ad-p53-associated genes, are primarily located in heterochromatin regions; **(E)** Comparison of gene expression (FPKM) of Ad-p53-associated genes between 5-FU treated and non-treated HCT116 p53 KO cells following Ad-p53 infection; (p-values by paired t-test, ns: *p* > 0.05, not significant); **(F)** Screenshots of BASIC browser views showing the expression of representative Ad-p53-associated genes, *HMGCR* and *USP53*, in Ad-p53 infected and uninfected KO cells, as well as WT cells, after 5-FU treatment for 0 h or 9 h.

To further explore the effect of Ad-p53 on host chromatin, we analyzed chromatin interactions between Ad-p53 and the host. The results demonstrated that p53 mediates chromatin interactions between Ad-p53 and various regions of the host chromosome (Figure 3A), with particularly high interaction frequencies observed on chromosome 17. Additionally, a slight increase in Ad-p53-host chromatin interactions was noted following 5-FU treatment.

To assess the impact of these interactions on host gene expression, we used the following criteria for analyzing Ad-p53-associated genes (Figure 3B). First, we filtered for high-confidence loops mediated by p53 and RNAPII, selecting those with more than 4 paired-end tag (PET) counts from Ad-p53 to host chromatin, and identified 6 Ad-p53-derived genes within the Ad-p53-host interaction regions. Next, we identified the Ad-p53-host interaction anchor regions on the host chromatin and filtered RNAPII mediated chromatin interactions that overlapped with these anchors. Based on the analysis of Ad-p53-associated host chromatin anchors and filtered RNAPII loop anchor regions, we identified 27 genes overlapping with these regions as Ad-p53-associated genes in the host. These genes were further classified based on their involvement in RNAPII or p53-mediated looping as p53 ChIA-PET genes, RNAPII ChIA-PET genes, or both. The interaction networks among these Ad-p53-associated genes are shown in Figure 3C.

We also investigated the chromatin state of Ad-p53-interacted regions in the host genome and their impact on gene expression. The analysis revealed that Ad-p53-interacted regions were predominantly located in heterochromatic and quiescent regions (Figure 3D). For comparison, we analyzed RNAPII top 1% (pileup value) peaks, which were primarily located in transcription start site (TSS)-associated regions (43.51%). Furthermore, RNAPII PET count top 1% loop anchors, p53 top 1% peaks, and p53 PET count top 1% loop anchors were more frequently located in TSS or enhancer regions. Since most Ad-p53 interaction regions are located in quiescent or heterochromatic regions, the expression levels of Ad-p53-associated genes showed no significant change after 5-FU treatment in Ad-p53-infected p53 KO cells (Figure 3E). Additionally, the expression of these Ad-p53-associated genes did not significantly change when compared to WT cells and p53 KO cells, regardless of 5-FU treatment (Figure 3F).

These findings indicate that, although Ad-p53 interacts with host chromatin, most interactions occur in quiescent or heterochromatic regions and do not affect the expression of Ad-p53-associated genes.

### 3.4 Ad-p53 does not impact host chromatin TADs

**FIGURE 4:**
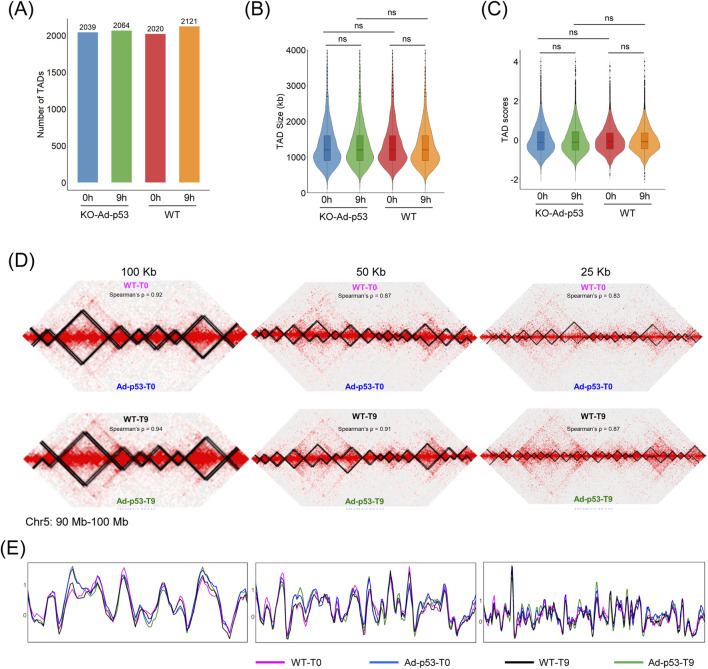
Ad-p53 does not impact host chromatin TADs. **(A)** TAD number of each treatment. T0: 5-FU untreated; T9: 5-FU treated for 9 h; **(B)** Violin plot of TAD size distributions across Ad-p53 infection and 5-FU treatments; **(C)** Violin plot of TAD boundary scores across Ad-p53 infection and 5-FU treatments (p-values by paired t-test, ns: *p* > 0.05, not significant); **(D)** 2D chromatin contact maps and TAD structure of HCT116 WT and p53 KO cells infected with Ad-p53 under 5-FU treated and untreated conditions, at resolutions of 100 kb, 50 kb, and 25 kb; **(E)** TAD insulation score comparison corresponding to **(D)**.

Although the Ad-p53 vector forms TAD-like structures and interacts with host chromatin, it remains unclear whether Ad-p53 affects the TAD structure in the host genome. To address this, we examined the TAD structure in p53 KO cells after Ad-p53 infection and compared it to HCT116 WT cells to exclude any effects caused by the p53 protein. Statistical analysis of the number of TADs showed no significant difference between Ad-p53-infected p53 KO cells and WT cells, regardless of p53 activation (Figure 4A). Additionally, no significant changes were observed in TAD size or TAD score across different treatments (Figures 4B, C).

We also compared TADs at resolutions of 100 kb, 50 kb, and 25 kb using Juicebox software. Representative screenshots of the chr5 (90 Mb–100 Mb) region showed that host TADs remained unchanged, regardless of Ad-p53 infection or 5-FU treatment (Figure 4D). TAD insulation score comparison curves were consistent across all treatments (Figure 4E). These results indicate that Ad-p53 infection and 5-FU treatment do not affect the host TAD structure.

### 3.5 The A/B compartments of the host chromatin were largely similar after Ad-p53 infection

**FIGURE 5:**
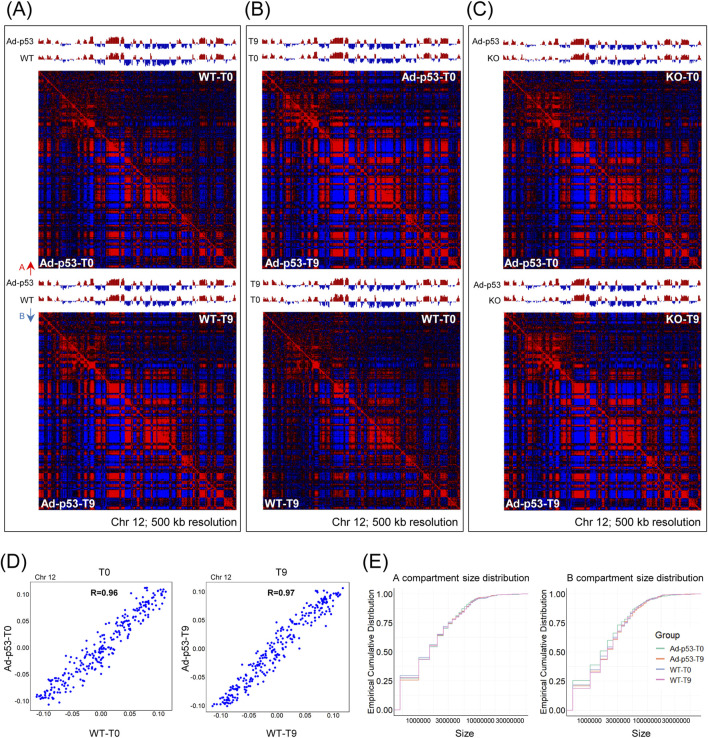
The A/B compartments of the host chromatin were largely similar after Ad-p53 infection. **(A)** Pearson correlation heatmap and A/B compartment eigenvector comparing HCT116 p53 KO cells infected with Ad-p53 and HCT116 WT cells under 5-FU-treated and untreated conditions. T0: 5-FU untreated; T9: 5-FU treated for 9 h. A/B compartments are represented by red and blue arrows; **(B)** Pearson correlation heatmap and A/B compartment eigenvector comparing 5-FU-treated and untreated conditions in p53 KO cells infected with Ad-p53 and WT cells; **(C)** Pearson correlation heatmap and A/B compartment eigenvector comparing p53 KO cells infected with Ad-p53 and p53 KO cells under 5-FU-treated and untreated conditions; **(D)** Scatterplots showing the correlation between the strength of A and B compartment calls for p53 KO cells infected with Ad-p53 and WT cells under 5-FU untreated (left) and 5-FU-treated (right) conditions. T0: 5-FU untreated; T9: 5-FU treated for 9 h. R = Pearson’s correlation coefficient; **(E)** The distribution of A (left) and B (right) compartment sizes across groups, assessed using the empirical cumulative distribution function.

To further investigate the effect of Ad-p53 on chromatin structure, we examined the compartment level. Pearson correlation heatmaps and A/B compartment eigenvectors were constructed. As shown in Figure 5A, we compared HCT116 p53 KO cells infected with Ad-p53 and HCT116 WT cells under both 5-FU-treated and untreated conditions. The A/B compartment structure and Pearson correlation heatmaps of the host genome remained largely unchanged.

We also compared 5-FU treated and untreated conditions in both p53 KO cells infected with Ad-p53 and WT cells (Figure 5B), and additionally compared Ad-p53-infected p53 KO cells to uninfected p53 KO cells (Figure 5C). All comparisons revealed consistent A/B compartment structures, with no significant changes in the Pearson correlation heatmaps. The compartment strength for Ad-p53 infected KO cells and WT cells were highly correlated both before and after 5-FU treatment (Figure 5D). Furthermore, we analyzed the A/B compartment sizes, and the results showed that compartment size distribution remained stable across different treatments (Figure 5E). Overall, the results indicate that the A/B compartment structure was mostly conserved and largely similar after Ad-p53 infection and p53 activation.

## 4 Discussion

The Ad-p53 represents a promising strategy for cancer therapy, yet its effects on host chromatin structure and gene expression remain insufficiently understood. This study provides a comprehensive analysis of the Ad-p53 vector’s 3D genome organization, its interactions with host chromatin, and its implications for host chromatin architecture and transcription regulation. By employing the ChIA-PET technique to map p53-and RNAPII-mediated chromatin interactions, along with RNA-seq to explore transcriptional outcomes, we elucidated the complex interplay between the Ad-p53 vector and host chromatin in colon cancer cells.

Collectively, our research demonstrates that p53 and RNAPII can bind to the Ad-p53 vector, mediating widespread chromatin interactions within the vector. Furthermore, we found that the Ad-p53 vector forms a unique 3D chromatin structure characterized by three block-like regions resembling TAD structures. Within these structures, p53-mediated highly conserved chromatin interactions, which likely contribute to its unique spatial organization. Such structural conservation within the Ad-p53 vector underscores the potential functional significance of vector architecture in gene regulation. Other studies also provide evidence that the adenovirus genome is not randomly positioned and exhibits structural features during infection in human cells (Giberson et al., 2012; Schwartz et al., 2023).

Consistent with findings from other studies on adenovirus interactions with host chromatin (Moreau et al., 2018; Kim and Lieberman, 2024), we also observed that the Ad-p53 vector interacts with host chromatin. Specifically, our study shows that Ad-p53 interactions with host chromatin predominantly occurred in quiescent or heterochromatic regions. This interaction pattern suggests a limited functional impact on host gene expression, as no significant transcriptional changes were detected in Ad-p53-associated host genes. Furthermore, the observation that the higher-order chromatin structures in host cells, including TADs and A/B compartments, remain largely unaltered during Ad-p53 infection and 5-FU-induced p53 activation reinforces the notion that the therapeutic effects of Ad-p53 are mediated independently of alterations in the global 3D architecture of the host genome.

In summary, our findings provide a comprehensive understanding of the Ad-p53 vector as a promising therapeutic tool for cancer treatment. Although Ad-p53 interacts with host chromatin, it does not alter the expression of associated host genes or disrupt the TADs and A/B compartment structures of the host genome. The therapeutic effects of Ad-p53 are likely primarily mediated by the p53 protein expressed from the vector, which is suggested to restore wild-type p53 function and regulate tumor suppressor pathways. These effects occur independently of any direct alteration to host chromatin architecture or gene expression by the Ad-p53 vector itself.

Moreover, our study provides valuable insights into the broader applications of adenovirus, such as vaccines based on adenoviral vectors (Park and Lee, 2024), and adenoviral oncolytic vector (Scarsella et al., 2024). These findings advance our understanding of how adenoviruses function, interact with host chromatin, and contribute to chromatin organization and gene regulation.

## Data Availability

The datasets presented in this study can be found in online repositories. The names of the repository/repositories and accession number(s) can be found below: https://www.ncbi.nlm.nih.gov/, PRJNA1196136.
